# The Novel Role of Metabolism-Associated Molecular Patterns in Sepsis

**DOI:** 10.3389/fcimb.2022.915099

**Published:** 2022-06-02

**Authors:** Xin-xu Zhu, Wen-wu Zhang, Cheng-hua Wu, Shun-shun Wang, Fang Gao Smith, Sheng-wei Jin, Pu-hong Zhang

**Affiliations:** ^1^ Department of Anaesthesia and Critical Care, The Second Affiliated Hospital and Yuying Children’s Hospital of Wenzhou Medical University, Zhejiang, China; ^2^ Academic Department of Anesthesia, Critical Care, Resuscitation and Pain, Heart of England NHS Foundation Trust, Birmingham, United Kingdom

**Keywords:** sepsis, metabolism-associated molecular patterns, free fatty acids, glucose, AGEs

## Abstract

Sepsis, a life-threatening organ dysfunction, is not caused by direct damage of pathogens and their toxins but by the host’s severe immune and metabolic dysfunction caused by the damage when the host confronts infection. Previous views focused on the damage-associated molecular patterns (DAMPs) and pathogen-associated molecular patterns (PAMPs), including metabolic proinflammatory factors in sepsis. Recently, new concepts have been proposed to group free fatty acids (FFAs), glucose, advanced glycation end products (AGEs), cholesterol, mitochondrial DNA (mtDNA), oxidized phospholipids (OxPLs), ceramides, and uric acid into metabolism-associated molecular patterns (MAMPs). The concept of MAMPs will bring new guidance to the research and potential treatments of sepsis. Nowadays, sepsis is regarded as closely related to metabolic disorders, and MAMPs play an important role in the pathogenesis and development of sepsis. According to this view, we have explained MAMPs and their possible roles in the pathogenesis of sepsis. Next, we have further explained the specific functions of different types of MAMPs in the metabolic process and their interactional relationship with sepsis. Finally, the therapeutic prospects of MAMPs in sepsis have been summarized.

## Overview: Metabolic Disorders in Sepsis

Sepsis is a highly heterogeneous and systemic inflammatory syndrome caused by an unbalanced host response to infection. The new definition of sepsis is a life-threatening organ dysfunction caused by a dysregulated host response to infection (Singer M. et al., 2016). Nowadays, sepsis is regarded as not only related to early infectious reactions but also related to cardiovascular (Coldewey et al., 2020), neurological (Yang Y. et al., 2019), biosynthetic (Sardari et al., 2021), metabolic, and other non-immune body reactions. Therefore, over time, the definition of sepsis has changed from a bacterial infection at the beginning to an inflammatory response and then to metabolic disorders in the body.

Sepsis can lead to severe and excessive inflammation. Our antioxidant and anti-inflammatory defense mechanisms help to balance the inflammatory stimulators and inhibitors. However, these systems can become overwhelmed and finally fail (Lauro and Limatola, 2020). The alteration of the immune system can contribute to metabolic abnormalities. Meanwhile, the disorder of immune metabolism can lead to a series of metabolic diseases such as obesity and diabetes (Muller et al., 2005; Falagas and Kompoti, 2006; Vachharajani and Vital, 2006; Frydrych et al., 2018). Metabolic disorders also cause cell death, chronic low-grade inflammation, and fibrosis ultimately (Hotamisligil, 2017). Damage-associated molecular patterns (DAMPs) are a group of different types of molecules derived from either the various components of the cell or the extracellular substance. In the situation of metabolic disorders, metabolism-derived DAMPs are increased significantly and are sensed by innate immune receptors, especially pattern recognition receptors (PRRs). Following interaction with PRRs and various non-immune receptors, DAMPs determine the downstream molecular signal resulting in sepsis (Schaefer, 2014; Patel, 2018).

## Novel Role of Metabolism-Associated Molecular Patterns in Sepsis

Metabolic disorders increase the risk of tissue damage and the number of endogenous dangerous signal molecules, which further deteriorate the metabolic disorders during sepsis (Christ and Latz, 2019). Metabolism-associated molecular patterns (MAMPs) are dangerous signal molecules derived from metabolic disorders and have been further classified to be a subset of DAMPs. In addition to free fatty acids (FFAs), glucose, advanced glycation end products (AGEs), and cholesterol, which have been regarded as members of MAMPs; some new members of MAMPs such as mtDNA, OxPLs, ceramides, and uric acid also have been identified (Wang et al., 2020). The proposal of MAMPs has a new guiding role for the research of sepsis and a potential clinical value for the treatment.

**Figure 1:**
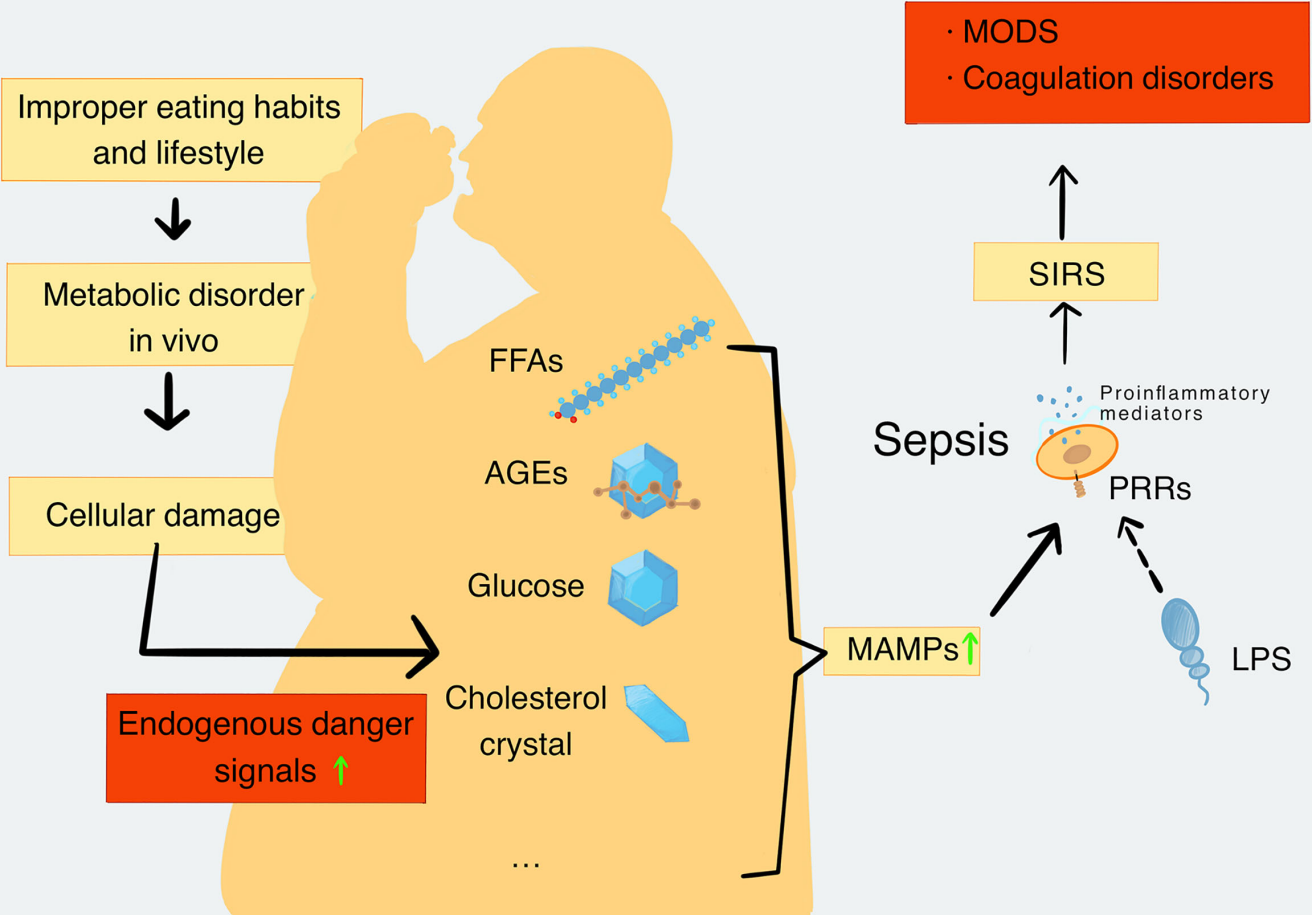
The production and types of MAMPs and their role in sepsis. When the metabolic balance is broken, the metabolic disorders will lead to a decrease in repair ability of the body. Meanwhile, metabolic disorders can cause extensive cell damage in the body tissues. Cell damage leads to increased levels of endogenous danger signaling molecules, including FFAs, AGEs, glucose, cholesterol crystal, and mtDNA, which are closely related to metabolic disorders and are defined as MAMPs. MAMPs can be recognized by PRRs and activate proinflammatory signaling pathways to promote the release of proinflammatory mediators. In addition, pathogenic substances such as LPS can also be recognized by PRRs and further activate the proinflammatory signaling pathway to cause the formation and deterioration of sepsis. Sepsis further enhances catabolism and increases MAMPs’ levels in turn. With the development of SIRS, the inflammatory process becomes uncontrollable, and the organ function and coagulation function become abnormal. Finally, these disorders cause the formation of MODS. MAMPs, metabolism-associated molecular patterns; FFAs, free fatty acids; AGEs, advanced glycation end products; mtDNA, mitochondrial DNA; PRRs, pattern recognition receptors; SIRS, systemic inflammatory response syndrome; MODS, multiple organ dysfunction syndromes.

Damaged cells release a number of endogenous risk signaling molecules such as excessive FFAs, glucose, cholesterol, AGEs, and mitochondrial DNA (mtDNA), which are regarded as MAMPs. They are sensed by innate immune receptors, particularly PRRs, which then activate proinflammatory signaling pathways and promote the release of proinflammatory mediators. The rise of proinflammatory mediators promotes metabolic inflammation and leads to chronic metabolic diseases eventually (Saltiel and Olefsky, 2017). In the later stages of the infection, inflammation becomes uncontrolled and leads to coagulation dysfunction, which is typical of the pathologic interaction between MAMPs and sepsis (Yang X. et al., 2019). In addition, numerous studies have also demonstrated that people with metabolic disorders such as obesity (Falagas and Kompoti, 2006; Vachharajani and Vital, 2006; Frydrych et al., 2018) or diabetes (Muller et al., 2005; Frydrych et al., 2018) have more risk of severe/fatal sepsis than lean people, further indicating the important role of MAMPs in sepsis (**Figure 1**). Therefore, explanations of various kinds of MAMPs and their possible roles in the pathogenesis of sepsis are important.

### Free Fatty Acid Metabolism in Sepsis

Fatty acids are the energy substrates of biological reactions and participate in related reactions of metabolism and synthesis. Long- and medium-chain fatty acids are produced through the decomposition of triglycerides (TG) in fat and liver tissue, while short-chain fatty acids are derived from indigestible dietary fiber produced by intestinal microbial fermentation (Kimura et al., 2020). Saturated fatty acids are classified as important MAMP types (Wang et al., 2020), which can induce inflammation by binding PRRs, particularly Toll-like receptors (TLRs) (Liang et al., 2018). Elevated FFA levels can be found in various metabolic disorders associated with diseases such as diabetes and obesity (Liang et al., 2018; Wong et al., 2019).

Clinical studies have also demonstrated an association between metabolic disorders of fatty acids and the systemic inflammatory response of sepsis. Serum TG levels are often elevated due to an increased total body fat oxidation and decreased TG hydrolysis in sepsis; the underlying reasons may be the dysregulation of the host response to lipopolysaccharide (LPS) (Weichselbaum et al., 2020). LPS and proinflammatory cytokines such as TNF-α, IL-6, and IL-1 rapidly induce the synthesis of FA and hepatic TG. TNF-α also can stimulate lipid accumulation in the liver, inhibit adipose tissue lipoprotein lipase, and decrease the clearance of lipoproteins. Meanwhile, high levels of endotoxin inhibit the activity of lipoprotein lipase, resulting in the elevation of plasma TG and then causing FFA levels to rise during sepsis (Weichselbaum et al., 2020). Elevated FFA induces systemic inflammation and insulin resistance leading to an increase in blood glucose levels (Plociennikowska et al., 2015). In addition, excess albumin-bound plasma non-esterified FFAs (NEFA) can leak through damaged glomeruli and be reabsorbed by renal proximal tubular cells, leading to proximal tubular cell damage (Sun H. et al., 2020). Furthermore, inhibiting adipogenesis can reduce inflammation and organ damage in sepsis (Idrovo et al., 2016). In brief, sepsis stimulates the production of FFA and elevates plasma FFA levels, which worsen the metabolic disorders and organ damage in turning.

### Cholesterol Metabolism in Sepsis

Cholesterol, one of the important MAMPs, plays an important role in the occurrence and development of metabolic diseases (Wang et al., 2020). Excessive activation of TLRs and inflammasome signals can lead to intracellular cholesterol accumulation and aggravate inflammatory responses (Tall and Yvan-Charvet, 2015). Cholesterol crystals, like HG, can also activate NLRP3 inflammasomes, which can activate the precursor of caspase-1 (pro-caspase-1), thereby promoting the maturation and release of the IL-1β family of cytokines and then mediating pyroptosis (He et al., 2016). Cholesterol crystals can also be sensed by the lectin and classical complement systems. Complement activation can be in crosstalk with the NLRP3 inflammasome pathway to aggravate the production of IL-1β (Niyonzima et al., 2017).

In patients with sepsis, the processes of microbial infection are closely related to cholesterol and lipids, and cholesterol also is involved in regulating the host’s inflammatory response (Paciullo et al., 2017). Lipid rafts are composed of proteins and lipids, which float freely within the liquid-disordered bilayer of cellular membranes but can also cluster to form larger, ordered platforms where functionally related proteins interact (Simons and Ehehalt, 2002). Cholesterol is an important component of the lipid rafts, and its role in lipid rafts is to act as a spacer among the hydrocarbon chains of sphingolipids and act as a dynamic glue to hold the lipid raft together (Borén et al., 2022). The lipid raft plays an important role in the conduction of inflammatory signals. After LPS stimulation, LPS-related receptor molecules in lipid rafts are activated, including CD14, heat shock protein (hsp) 70 and 90, chemokine receptor 4 (CXCR4), growth differentiation factor 5 (GDF5), and TLR4. The activation and enhancement of inflammatory signals are also closely related to the enrichment of cholesterol in lipid rafts in turning (Radermecker et al., 2019). Clinical studies have shown that some cholesterol-lowering drugs, such as statins, can enhance the immune suppression response mediated by regulatory T cells (T-regs) and can reduce the damage caused by systemic inflammation simultaneously in sepsis (Needham et al., 2016). In short, cholesterol participates in sepsis-mediated inflammation and organ damage, and controlling and reducing the levels of plasma cholesterol may be necessary for sepsis treatment.

### Glucose Metabolism in Sepsis

Glucose is also regarded as a typical MAMP (Wang et al., 2020). High glucose (HG) is considered to be a major endogenous danger signal for diabetes and its complications. HG induces proinflammatory cytokines production *via* PRRs in a variety of cell populations such as macrophages, adipocytes, and muscle cells (Pahwa et al., 2016). During the pathogenesis of insulin resistance, diabetes, and diabetic complications, persistent hyperglycemia activates NOD-like receptors (NLRs), particularly the NLRP3 inflammasomes, which can promote the inflammatory response (Wang et al., 2020). HG also stimulates the expression of thioredoxin-interacting protein and activates ATP/P2X4 signaling, both of which lead to the activation of NLRP3 inflammasomes, causing severe metabolic inflammation (Han et al., 2019). In addition to activating NLRs, HG has been shown to induce the activation of TLR4-dependent signaling, which promote kidney and heart inflammation (Han et al., 2019).

Pyroptosis is considered a pathway of inflammatory programmed cell death. NLRP3 inflammasomes can cleave pro-caspase-1 into activated caspase-1, promote the formation of mature IL-1β and IL-18 precursors, and then mediate pyroptosis and plays an important role in the development and maintenance of inflammatory response (He et al., 2016). Under HG and hypoxia/reoxygenation (H/R) conditions, NLRP3 inflammasome-induced pyroptosis and inflammatory pathways are activated and can be further enhanced by LPS stimulation (Qiu et al., 2019).

**Figure 2:**
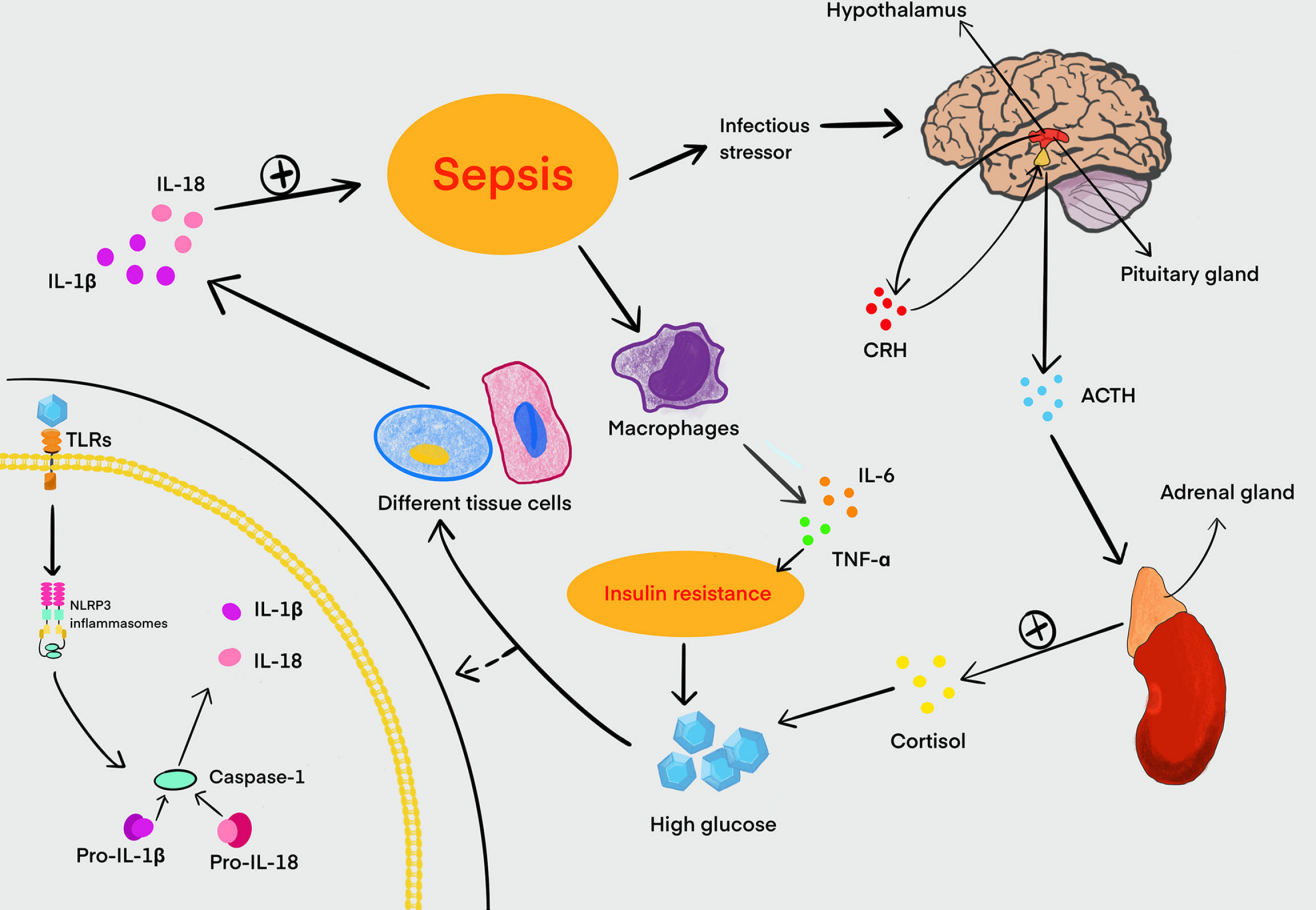
Relationship between HG and sepsis. Sepsis can stimulate the hypothalamus to increase the secretion and release of CRH. Meanwhile, CRH promotes the synthesis and release of ACTH from the pituitary gland, and ACTH in turn stimulates the adrenal cortex to secrete cortisol, which can lead to an increase in blood glucose. Sepsis can also stimulate macrophages to secrete large amounts of IL-6, TNF-α, and other proinflammatory mediators. These proinflammatory mediators can disrupt the signaling of the insulin transduction pathway, and it is a major cause of insulin resistance in the host. Insulin resistance can further exacerbate HG in turning. Glucose is recognized by TLRs in a variety of tissue cells and activates NLRP3 inflammasomes that can cleave pro-caspase-1 into activated caspase-1 to induce inflammation. Activated caspase-1 processes pro-IL-1β and pro-IL-18 to form mature IL-1β and IL-18, promoting the development of inflammation in sepsis. HG, high glucose; CRH, corticotropin-releasing hormone; ACTH, adrenocorticotropic hormone; IL, interleukin; TNF, tumor necrosis factor; TLRs, Toll-like receptors.

Studies have also shown that LPS can disturb the homeostasis of blood glucose, and this effect may be related to the release of glucagon and insulin (Wang et al., 2013; Qiu et al., 2019). In sepsis, abnormal blood glucose is often manifested as hyperglycemia or hypoglycemia, which can cause organ function damage. It is generally believed that HG is one of the responses related to the systemic inflammatory response during sepsis. Sepsis also can promote the secretion of cortisol by activating the hypothalamic–pituitary–adrenal axis to induce an increase in blood glucose (Pivonello et al., 2016). The systemic inflammatory response of sepsis leads to the release of a number of proinflammatory mediators such as IL-6 and TNF-α, which induce the production of cytokine signaling inhibitors. These inhibitors can damage the signaling of the insulin transduction pathway, cause the downregulation and translocation impairment of GLUT3, and lead to insulin resistance finally. This is also the main cause of sepsis hyperglycemia (Pivonello et al., 2016). Persistent HG also activates NLRP3 inflammasomes, which further aggravate the inflammatory response in turn. Therefore, sepsis patients with hyperglycemia have a higher mortality rate (Muller et al., 2005; Frydrych et al., 2018). Interestingly, hypoglycemia also can aggravate the severity of sepsis and increase the mortality of sepsis patients simultaneously (Investigators et al., 2012). Glucose metabolism is closely associated with the liver, and hypoglycemia has been reported as a common manifestation of sepsis in patients with cirrhosis, which may cause the inhibition of gluconeogenesis under the condition of glycogen consumption (Maitra et al., 2000) (**Figure 2**). Hence, neither hyperglycemia nor hypoglycemia is beneficial to the recovery and prognosis of sepsis.

### Advanced Glycation End Product Metabolism in Sepsis

AGEs are formed with the interaction between aldose and proteins or lipids, and the subsequent molecular rearrangement of covalently linked glucose. The production of AGEs, as endogenous metabolites, is increased in patients with hyperglycemia (Wang et al., 2020). AGEs exist in plasma and accumulate in the vessel walls and tissues along with the aging process. The formation and deposition of AGEs are significantly increased in hyperglycemic patients. The degree of oxidative stress is also a key factor in the formation of AGEs (Wang et al., 2020).

Several receptors of AGEs (RAGE) have been identified, and activating the RAGE can mediate a series of cellular responses. Like MAMP, AGEs can directly activate the TLR2/4 signaling pathway in various cells and stimulate the production of proinflammatory cytokines such as IL-6 and TNF-α to take part in the pathogenesis of sepsis (Koch et al., 2010; Zhang et al., 2017). Innate immune response in sepsis is inseparable from RAGE (Hofer et al., 2016). Studies have found that both RAGE and its ligand AGEs on the surface of neutrophils of peripheral blood were significantly increased in sepsis patients (Forero-Pena and Gutierrez, 2013). Therefore, RAGE participates in the occurrence and development of sepsis through the interaction of innate immune cells.

### MtDNA Metabolism in Sepsis

Mitochondria are the energy centers of cell activity and promote the body’s metabolism by producing ATP. Human mtDNA is a naked circular double-stranded DNA molecule whose full length is about 165–168 bp. MtDNA shares many similarities with bacterial DNA (bDNA) and is rich in unmethylated CpG repeat motifs. Studies have shown that CpG repeat motifs are the structures in which bDNA bind PRRs and produces inflammatory effects (Zhang et al., 2010). Currently, most believe that mtDNA, which is similar to bDNA in the basic structure, can promote the development of sepsis by activating TLR9/NLRP3 signaling pathways. Of note, the exact mechanisms require more research. In the case of metabolic disorders, excess nutrients (such as glucose and FFA) can induce mitochondrial damage and dysfunction (Munoz and Costa, 2013). Mitochondrial dysfunction has been demonstrated to be closely associated with the occurrence and development of sepsis. The levels of free mtDNA in plasma are positively related to the risk of organ dysfunction during sepsis. Mechanically, in the TLR9 signaling pathway, mtDNA binds to the TLR9 of neutrophils to stimulate neutrophils to release measuring matrix metalloproteinase (MMP)-8 and MMP-9 and produce TNF-α, IL-1, IL-6, and other proinflammatory factors. Furthermore, in the NLRP3 signaling pathway, mtDNA is released into the cytoplasm after mitochondrial injury, then induces the activation of the NLRP3 inflammasome, and contributes to the secretion of IL-1β and IL-18 (Liao et al., 2022).

### Other Metabolism-Associated Molecular Patterns in Sepsis

Other MAMPs such as oxidized phospholipids (OxPLs), ceramides, and uric acid also play an important role in sepsis (Wang et al., 2020). OxPLs are potential participants in acute and chronic inflammation. Studies have found that OxPLs not only are produced in the inflammatory response but also have a regulatory effect on inflammation. OxPLs are an important proinflammatory mediator in atherosclerosis (Que et al., 2018) and non-alcoholic steatohepatitis (Sun X. et al., 2020). However, recent studies have found that in addition to their proinflammatory effects, OxPLs also have powerful anti-inflammatory properties. OxPLs can inhibit LPS-induced upregulation of inflammatory genes by inhibiting the interaction of LPS with LPS-binding protein and CD14, but OxPLs do not inhibit TNF-α-induced or IL-1β-induced upregulation of NF-κB-mediated inflammatory genes. Therefore, the discovery of chemical structures that inhibit the effect of LPS is of great significance for the treatment of sepsis. Acid sphingomyelinase is activated by a number of pathogens and plays a harmful role in the development of apoptosis and organ failure in sepsis (Peng et al., 2015). The increased concentration and activity of acid sphingomyelinase in plasma indicate the severity of the disease in patients with sepsis (Chung et al., 2016). Acid sphingomyelin then mediates the release of ceramides, which play an important role in apoptosis. Recent studies suggest that ceramides are related to a variety of metabolic disorders (Summers, 2018). Ceramides mediate the formation and activation of the NLRP3 inflammasome under different pathological conditions, can activate caspase-1, and promote the release of IL-1β and other cytokines (He et al., 2016). Uric acid is the end product of purine metabolism during the normal physiological process. Uric acid can be involved in the inflammatory response by activating the renin–angiotensin system. In patients with sepsis, elevated serum uric acid levels may be associated with systemic inflammation (Kir et al., 2021). Uric acid crystals can promote inflammation by activating NLRP3 inflammasomes, and soluble uric acid can induce CCL2 production to drive monocyte aggregation (Wang et al., 2020). The causes of elevated uric acid crystal levels are related to metabolic disorders of sepsis rather than proximal tubular injury (Tabibzadeh et al., 2021). Cell death not only can release the stored uric acid but also can produce huge quantities of uric acid due to nucleic acid degradation, while uric acid has been proved to be an important proinflammatory molecule (Tabibzadeh et al., 2021).

## Therapeutic Prospects of Metabolism-Associated Molecular Patterns in Sepsis

Although it is now recognized that sepsis is closely related to metabolic disorders and can lead to persistently excessive inflammation, the treatment of sepsis still remains a difficult task. Metabolic disorders can lead to high levels of MAMPs. Therefore, further research on the role of MAMPs in sepsis is important for the therapeutic prospects of sepsis. For example, OxPLs also have anti-inflammatory effects, and OxPLs contain chemical structures that inhibit LPS activation (Oskolkova et al., 2021). Recent studies have shown that the oxidized1-palmitoyl-2-arachidonoyl-sn-glycero-3-phosphorylcholine (PAPC), but not the non-oxidized PAPC, significantly inhibited LPS-induced TNF-α response (Oskolkova et al., 2017). This suggests that drugs that contain compounds with protective barrier properties have great potential in the treatment of sepsis.

Further studies of MAMPs and their associated signaling pathways are also highly anticipated in the treatment of sepsis. MAMPs play an important role in promoting the activation of inflammatory pathways. Searching for or preparing drugs that can inhibit the rise of some MAMPs levels or inhibit the pathway response mediated by MAMPs may be a promising treatment for sepsis.

## Conclusion

Today, sepsis is still the main cause of death in intensive care unit patients, and effective treatment means are very scarce. Although some treatments have been proposed, few appropriate treatments have been successfully applied in the clinic due to severe side effects or low efficacy (Aliomrani et al., 2016; Singer B.H. et al., 2016). The proposal of the concept of MAMPs brings a new standard for the classification of inflammatory stimulation, provides a more accurate description of metabolic inflammation, and further assists the study of the pathogenesis of sepsis. Here, we provide a preliminary summary of the role of these MAMPs’ metabolism in the pathogenesis of sepsis, and it is believed that MAMPs have considerable therapeutic prospects for sepsis. Although the relationship between MAMPs and sepsis is inseparable, there are still some potential problems that need to be solved. Firstly, the current discovery of MAMPs is far from complete, and new MAMPs will be continuously explored, as they may be also involved in the inflammatory process or induce the inflammatory response in sepsis. Secondly, the detailed mechanisms of action of various MAMPs in sepsis are not comprehensive and in-depth, and the relationship between MAMPs and sepsis is worthy of further research. Finally, treatment of sepsis with MAMPs will also be a potential problem, and there may be a long way to search for a safe and reliable treatment strategy for sepsis.

## Funding

This study was supported by the National Natural Science Foundation of China (No. 81571862, No. 81870065, and No. 82070082), China Postdoctoral Science Foundation (2021M702502), and Youth Scientific Research Innovation Project of Wenzhou Medical University (KYYW202130).

## Conflict of Interest

The authors declare that the research was conducted in the absence of any commercial or financial relationships that could be construed as a potential conflict of interest.

## Publisher’s Note

All claims expressed in this article are solely those of the authors and do not necessarily represent those of their affiliated organizations, or those of the publisher, the editors and the reviewers. Any product that may be evaluated in this article, or claim that may be made by its manufacturer, is not guaranteed or endorsed by the publisher.
